# Affective flexibility as a developmental building block of cognitive reappraisal: An fMRI study

**DOI:** 10.1016/j.dcn.2022.101170

**Published:** 2022-10-31

**Authors:** Jordan E. Pierce, Eisha Haque, Maital Neta

**Affiliations:** Cognitive and Affective Neuroscience Laboratory, Center for Brain, Biology, and Behavior, University of Nebraska-Lincoln, Lincoln, NE, USA

**Keywords:** Flexibility, Cognitive reappraisal, Emotion regulation, Ambiguity, Development, FMRI

## Abstract

Cognitive reappraisal is a form of emotion regulation that involves reinterpreting the meaning of a stimulus, often to downregulate one’s negative affect. Reappraisal typically recruits distributed regions of prefrontal and parietal cortex to generate new appraisals and downregulate the emotional response in the amygdala. In the current study, we compared reappraisal ability in an fMRI task with affective flexibility in a sample of children and adolescents (ages 6–17, N = 76). Affective flexibility was defined as variability in valence interpretations of ambiguous (surprised) facial expressions from a second behavioral task. Results demonstrated that age and affective flexibility predicted reappraisal ability, with an interaction indicating that flexibility in children (but not adolescents) supports reappraisal success. Using a region of interest-based analysis of participants’ BOLD time courses, we also found dissociable reappraisal-related brain mechanisms that support reappraisal success and affective flexibility. Specifically, late increases in middle prefrontal cortex activity supported reappraisal success and late decreases in amygdala activity supported flexibility. Together, these results suggest that our novel measure of affective flexibility – the ability to see multiple interpretations of an ambiguous emotional cue – may represent part of the developmental building blocks of cognitive reappraisal ability.

## Highlights

• Cognitive reappraisal is a crucial affective function that develops in childhood.
• Affective flexibility of valence judgments supports reappraisal success in children.
• Increased late PFC activity supports reappraisal success.
• Decreased late amygdala activity supports affective flexibility.
• Flexibility may be one developmental building block of emotion regulation ability.

## 1 Introduction

Emotion regulation is a critical affective process by which individuals can modulate their own physiological and subjective responses to biologically relevant stimuli (Buhle et al., 2014, Gross, 2015, McRae et al., 2012). Cognitive reappraisal is one common form of emotion regulation that involves reinterpreting the meaning of a stimulus, often to downregulate one’s negative affect. When implemented in daily life, effective reappraisal is typically beneficial to one’s psychological well-being (Gross and John, 2003, McRae et al., 2012). Functional magnetic resonance imaging (fMRI) studies have shown that cognitive reappraisal is supported by several brain regions including various areas of prefrontal cortex (PFC) that exert cognitive control over affective regions such as the amygdala (Buhle et al., 2014, Frank et al., 2014, Ochsner et al., 2012).

Emotion regulation abilities emerge during childhood (Rydell et al., 2003, Sala et al., 2014) and continue to improve through adolescence into adulthood, in parallel with PFC maturation and cognitive control abilities (Luna et al., 2015, McRae et al., 2012). Accordingly, the recruitment of emotion regulation-related brain regions varies as a function of individual differences such as age and reappraisal ability (McRae et al., 2012, Pierce et al., 2022, Pitskel et al., 2011). Children typically exhibit the weakest PFC response during emotion regulation, associated with worse reappraisal effectiveness and poorer cognitive control (McRae et al., 2012). The strength of the PFC response and cognitive reappraisal abilities then increase over development (McRae et al., 2012, Silvers et al., 2017). In contrast, children and adolescents often have stronger amygdala reactivity to emotional stimuli compared to older participants (Pitskel et al., 2011, Silvers et al., 2015, Stephanou et al., 2016).

The maturation of emotion regulation is supported by the development of various processes that contribute to this multifaceted affective function. One crucial mental process that supports reappraisal is the ability to flexibly interpret or attend to distinct aspects of affective stimuli (Gross, 2015, Ochsner et al., 2012, Schweizer et al., 2020). This affective flexibility allows an individual to more readily create alternate appraisals of the stimulus or (dis)engage with certain aspects of a situation that may impact its emotional meaning (Hollenstein, 2015, Malooly et al., 2013, Zhu and Bonanno, 2017). Affective flexibility has been characterized in several ways including the ability to switch between emotional and non-emotional task sets (Genet et al., 2013, Grol and De Raedt, 2018, Malooly et al., 2013, Samson et al., 2022, Twivy et al., 2021) and variability in one’s emotional states (Hollenstein, 2015, Koval et al., 2013). Previous research that defined affective flexibility as a task switching ability has reified its relationship with emotion regulation in adults (Malooly et al., 2013): those who could more readily disengage from emotional stimuli were also more capable of reinterpreting images as less negative.

Another crucial process that supports the development of emotion regulation is cognitive control, which adapts responses to meet current situational demands (Buhle et al., 2014, Malooly et al., 2013). Adults, and to a lesser degree adolescents, rely on cognitive control resources to enact reappraisal (McRae et al., 2012). In contrast, given children’s underdeveloped cognitive control skills (Luna et al., 2015, McRae et al., 2012), they may engage alternative skills to achieve optimal performance. Affective flexibility may be one such skill, yet little work has linked flexibility to emotion regulation ability in development. The studies that have examined this relationship (e.g., Martins et al., 2020; Schweizer et al., 2020; Tottenham et al., 2011) often used tasks that conceptualized affective flexibility as an attentional shift between emotional and non-emotional material, which – to some extent – is conflated with cognitive control. For example, in one study of affective flexibility (Mărcuş et al., 2016), children (ages 11–14 years) completed a matching task using shapes or emotional faces that required flexible shifting between stimulus features. The youngest participants were slowest to respond across all trials but more so when categorizing *emotional* rather than non-emotional stimuli. It remains unclear, however, how well children can demonstrate affective flexibility when the task is less dependent on cognitive control.

We recently introduced a novel measure of affective flexibility (Pierce et al., 2022) that is operationalized as variability in valence judgments of ambiguous emotional faces. In our task, participants make a forced-choice decision categorizing angry, happy, and surprised faces as having either negative or positive valence (Kim et al., 2003, Neta et al., 2009, Petro et al., 2021). Angry and happy faces have a relatively clear valence that should be interpreted as negative and positive, respectively, across all participants. Surprised faces, in contrast, are considered ambiguous in that they can signal either positive or negative valence in different contexts (e.g., an unexpected gift or news of a tragedy). In our task, stimuli are presented without contextual information and an individual’s valence judgments arise largely from their own internal affective bias (i.e., "valence bias," Neta et al., 2009). The variability with which one interprets surprised faces, therefore, represents flexibility in one’s affective appraisals. Notably, this task is developmentally appropriate and does not rely on cognitive control resources, as does, for example, inhibiting emotional processing in a go/no-go task (e.g., Tottenham et al., 2011).

Using this novel measure of affective flexibility, we found an age-related difference in the link between flexibility and reappraisal success (Pierce et al., 2022): children (ages 6–11 years) with better affective flexibility showed greater reappraisal success (adolescents and adults showed no relationship). To better understand the mechanism linking affective flexibility and cognitive reappraisal in development, the current study examined the neural correlates of these processes in a cognitive reappraisal fMRI task using emotional scenes in a sample of 76 children and adolescents (6–17 years old). We extracted hemodynamic time courses in 11 reappraisal-related regions of interest (ROIs; Petro et al., 2018) for reappraise and control trials and examined the relationship between these brain responses in early and late trial time windows and our behavioral measures (affective flexibility, reappraisal success). Based on prior emotion regulation findings in young adults (McRae et al., 2010, Pierce et al., 2022) and children (McRae et al., 2012, Silvers et al., 2015), we hypothesized that greater activation in prefrontal ROIs and reduced activation in amygdala ROIs would predict greater affective flexibility and better reappraisal success.

## 2 Methods

### 2.1 Participants

One hundred and sixteen participants between the ages of 6 and 17 were recruited via community flyers for a two-session study (these same participants are included within the larger sample in Pierce et al., 2022). All participants were right-handed and reported no history of neurological or psychiatric disorder. Of this initial group, 15 were removed due to inaccurate ratings on clear valence trials (see below) or because of MRI exclusion criteria (e.g., braces). The remaining 101 individuals were invited to participate in a second session in the MRI. Of those, eight participants failed to complete the entire emotion regulation task, two participants were excluded for neuroanatomical irregularities, seven participants were excluded due to excessive motion during the functional scans, and behavioral data from another eight were not recorded due to a technical error. This left a final sample size of 76 participants (mean age = 11.34 years (SD = 2.97)), comprising 37 males and 39 females, who reported their race as 71.1% White/non-Hispanic, 11.8% White/Hispanic, 5.3% Black, 9.2% more than one race/non-Hispanic, and 2.6% more than one race/Hispanic. This sample size is consistent with comparable studies in the literature (e.g., Gee et al., 2013; McRae et al., 2012; Silvers et al., 2015) and our initial recruitment goals given practical limitations on fMRI data acquisition. All children provided verbal assent with parent/guardian written consent. Participants received monetary compensation for each session and all study procedures were approved by the UNL Institutional Review Board.

### 2.2 Task design and procedure – Session 1

In the first behavioral session, participants performed a task to assess their affective flexibility in which they viewed images of positive (happy), negative (angry), and ambiguous (surprised) facial expressions on a white background (see Pierce et al., 2022 for a full description). For each image, participants were asked to make a two-alternative, forced-choice decision, using the computer mouse, indicating whether each image felt “good” or “bad”. Participants were instructed to respond quickly but accurately. The stimuli consisted of a set of 48 White adult faces, 24 with an ambiguous valence (surprised expression), and 24 with a clear valence (12 angry and 12 happy expressions) from the NimStim Set of Facial Expressions (Tottenham et al., 2009) and the Karolinska Directed Emotional Faces database (Goeleven et al., 2008), which were selected for having a hit rate > 60% for emotion identification in the original studies. These facial expressions were presented in blocks that alternated with blocks of scenes from the International Affective Picture System (IAPS; Lang et al., 1997), consisting of 24 scenes with an ambiguous valence and 24 with a clear valence (12 negative and 12 positive). The purpose of rating these IAPS scenes is outside the scope of the current report and did not contribute to the flexibility score.

Stimuli were presented and responses collected using MouseTracker software (Freeman and Ambady, 2010); only the response choice is analyzed here. Trials were self-paced and began with the presentation of a fixation cross for 1000 ms, followed by a face stimulus for 1000 ms. The response options appeared, along with the face, in the upper left and right corners of the screen and remained visible after the face presentation until a response was made. If a response was not initialized within 2000 ms of the stimulus onset, a screen appeared instructing the participant to initiate a movement more quickly. As in prior work (e.g., Neta et al., 2013; Neta and Whalen, 2010; Petro et al., 2021), participants were excluded if their judgments of angry and happy faces were below 60% accuracy as this could indicate that they did not understand or were not attending to the task. All others who met inclusion criteria were invited to return for an MRI session about one week later.

### 2.3 Task design and procedure – Session 2

During the second session, participants completed an emotion regulation task in the MRI (see Pierce et al., 2022 for a full description). Participants were positioned on their back in the scanner and viewed the task screen via a mirror attached to the head coil. Briefly, each trial began with an instruction screen presented for 2000 ms, with either “Look” or “Decrease” written on a green or blue background, respectively, followed by the 7000 ms presentation of an emotional IAPS image. For the trials with a look instruction, half of the images had negative valence (“look negative”) and half of the images had neutral valence (“look neutral”). In these control conditions, participants were instructed to respond naturally and allow whatever feelings may arise when viewing the image, without trying to modify their emotions. For the trials with a decrease instruction (“reappraise”), all the images were negative and participants had to cognitively reinterpret the content to make themselves feel less negative. They were instructed that reinterpretation could consist of reappraisals such as “imagine the scene is from a movie” or “help will soon arrive”, but that their attention should remain on the content of the image and not involve looking away or mental distraction. Next, a 4000 ms rating screen appeared where participants had to indicate the degree of negative emotion felt after viewing each image: “How bad do you feel?” on a scale from 1 (not at all) to 5 (very bad). Finally, there was a “Rest” screen that lasted 1000, 2000, or 3000 ms before the next trial began.

Participants first completed a set of three practice trials. The task itself consisted of 20 trials each of look negative, look neutral, and reappraise trials pseudo-randomly distributed throughout the task (60 total trials, all with unique images that were not shown during practice or in the scenes task in Session 1). Stimulus order was counterbalanced across participants and the task was presented using E-Prime software (Psychological Software Tools, Inc., Pittsburgh, PA). Responses were recorded via an MR-compatible button box. An anatomical scan was collected first, followed by two passive face viewing functional scans, the emotion regulation task, and finally a resting-state scan (only the regulation task will be described here).

### 2.4 MRI acquisition parameters

Scanning was performed at the Center for Brain, Biology, and Behavior (CB3) at UNL on a Siemens 3 T Skyra scanner using a 32-channel head coil. Structural images were collected using a T1-weighted MPRAGE sequence with the following parameters: 192 interleaved slices, TR = 2200 ms, TE = 3.37 ms, voxel size = 1.0 mm^3^, matrix = 256 × 256, FOV = 256 mm^2^, flip angle = 7°. For the functional tasks, blood oxygen level-dependent (BOLD) activation was measured with an EPI sequence with the following parameters: 51 interleaved slices, multiband acceleration factor = 3, TR = 1000 ms, TE = 29.8 ms, voxel size = 2.5 mm^3^, matrix = 84 × 84, FOV = 210 mm^2^, flip angle = 60°, 474 volumes, total acquisition time = 8:08 per run. Slices were acquired parallel to the AC-PC plane.

### 2.5 Data analysis

Positive and negative responses on surprise trials from the valence bias task were combined within participant to create a flexibility score (0–100 possible range). This score was calculated as 100 x (1- (abs(Neg – Pos)/Neg + Pos)), with Neg = number of negative responses on surprise trials and Pos = number of positive responses on surprise trials, as in prior work (Pierce et al., 2022). Thus, participants with greater variability in their judgments of surprise across trials were scored as more flexible (e.g., individuals with an equal number of positive and negative responses received a 100% flexibility score) and those with lower variability were scored as less flexible (e.g., those with all positive or all negative responses received a 0% flexibility score). Given that participants with inaccurate ratings for angry or happy faces were excluded, high variability for surprised faces should reflect true flexibility in valence judgments of ambiguity rather than a random, inattentive response pattern.

For the emotion regulation task, participants’ reappraisal success scores were calculated as the ratings for “look negative” trials minus the ratings for “reappraise” trials, multiplied by the “look negative” ratings (as in Petro et al., 2018; Pierce et al., 2022) to account for potential individual differences in initial reactivity to the negative images. A linear regression model was fit with reappraisal success as the dependent variable and predictors of age (as a continuous variable), valence bias, flexibility, and the interaction of age x flexibility. Valence bias (overall proportion of negative vs. positive judgments) was included to control for any effects of internal affective bias that might influence the relationship between flexibility and reappraisal.

Functional data were analyzed using the AFNI software package (Cox, 1996, Cox, 2012). Preprocessing included de-spiking of time series outliers, slice timing correction, alignment of functional volumes to each other and the individual anatomical image, standardization to the Talairach atlas space (Talairach and Tournoux, 1988), smoothing with a 6-mm FWHM kernel, and scaling of each voxel to a mean of 100. Next, the data were entered into a regression model (3dREMLfit) with regressors for each trial type (reappraise, look negative, look neutral) using the “TENT” function to estimate the amplitude of the hemodynamic response at each TR from 0 to 16 s after stimulus onset (17 timepoints; TR = 1 s) without assuming a predetermined shape for the response in each voxel. 3dREMLfit uses a generalized least squares approach to estimate the temporal auto-correlation in the time series using an ARMA(1,1) model. Regressors of no interest included polynomials for each run (four terms) to account for slow drift in the time series and twelve motion parameters (x, y, z shift/rotation estimated during alignment and their derivatives). Look neutral trials were included in the task to minimize habituation effects and were not of interest in the current analysis.

Subsequently, the beta values at each time point (i.e., the estimated hemodynamic response function (HRF)) were extracted for each trial type from 11 regions of interest (ROIs) based on a previous study of emotion regulation using reappraisal of negative images (Petro et al., 2018). Here, each ROI was defined as a 6-mm sphere centered on the peak coordinates at each location in the previous study’s reappraise > look negative contrast, as well as left and right amygdala for which they reported greater activation for look negative than reappraise trials. Consistent with our previous analysis in adults (Pierce et al., 2022), difference values (reappraise-look negative) were calculated at each point in the time course for each ROI and then averaged within early (4–8 s post-stimulus onset) and late (11–15 s post-stimulus onset, i.e., 4–8 s post-rating onset) phases of the trial. These windows were selected to capture activation in the time around the potential peak responses following the onset of the emotional image or rating screen, given that the HRF typically peaks about 5–6 s after stimulus onset (Miezin et al., 2000). One sample *t*-tests (two-sided vs. zero) were conducted on the average activation differences from the two time-windows in each ROI, using Holm’s adjusted *p*-values to account for multiple comparisons.

Next, the behavioral measures of reappraisal success and affective flexibility were correlated with the averaged peak activation from four groups of ROIs, controlling for the effect of age: a) those with a significant or trend-level increase in the early window (3 ROIs), b) those with a significant or trend-level increase in the late window (1 ROI), c) the bilateral amygdala in the early window (2 ROIs), and d) the bilateral amygdala in the late window (2 ROIs). Averaged activation across ROIs was used based on the assumption that emotion regulation regions that respond similarly (increased/decreased activation) are contributing cooperatively to behavioral outcomes, and to reduce the number of statistical tests being run. (Note that no regions showed a significant decrease in either the early or late windows.) Although activation in the amygdala was not significantly decreased during reappraisal in this sample, we chose to include it as a variable of interest given prior findings in this region during emotion regulation (e.g., Buhle et al., 2014; Pierce et al., 2022; Silvers et al., 2017). Specifically, our previous study with the same emotion regulation task using a similar analysis (Pierce et al., 2022), identified a relationship between late amygdala activation and reappraisal. Significance levels were set at *p* < .05 using Holm’s adjusted *p*-values to correct for multiple comparisons, and Spearman correlations (*ρ*) were used due to the non-normal distribution of flexibility (Shapiro-Wilk test *W*=0.934, *p* < .001). Statistical analyses were performed using SPSS version 28 (IBM, Cary, NC, USA).

## 3 Results

### 3.1 Behavior

Flexibility was calculated based on the variability in valence responses for ambiguous (surprised) faces, with smaller values indicating lower variability in judgments (e.g., always negative) and larger values indicating higher variability (e.g., equal number of positive and negative judgments). The mean flexibility score was 44.52 (SD = 31.43, range: 0–100). For the emotion regulation task, consistent with prior work (Petro et al., 2018, Pierce et al., 2022), an individual’s reappraisal success was calculated as (look negative-reappraise trial rating)*look negative rating, with larger positive values indicating a greater reduction in negativity during reappraisal, scaled by the extent of negativity experienced when not regulating (possible range: −6.25 to 20). The mean reappraisal success in our sample was 3.96 (SD = 3.64, range: −6.12 to 11.36).

The linear regression model predicting reappraisal success was significant overall (*F*(4,71)= 3.158, *p* = .019, *R*^2^ = .151) and included significant effects of age, flexibility, and the interaction of age and flexibility, with a non-significant effect of valence bias (Table 1). The age and flexibility main effects indicated that older and more flexible participants had better reappraisal success. Further examination of the interaction effect indicated that the slope of flexibility was significant in the region from ages 6.00–10.91 years and non-significant from ages 10.91–17.00 years. The simple slope of flexibility was positive in the region of significance, indicating that for the younger participants greater affective flexibility was associated with better reappraisal.

**Table 1:** Behavior linear regression model description.

	*B*	95% C.I.	*β*	*t*	*p*	*r*_*a (b.c)*_
*Reappraisal Success*						
Constant	-5.119	[− 10.643, 0.406]		-1.848	0.069	
Age	0.704	[0.236, 1.172]	0.576	2.999	0.004**	0.328
Valence Bias	0.338	[− 2.180, 2.856]	0.029	0.267	0.790	0.029
Flexibility	0.131	[0.026, 0.235]	1.128	2.495	0.015*	0.273
Age*Flexibility	-0.010	[− 0.018, − 0.001]	-1.053	-2.163	0.034*	-0.237

B: unstandardized coefficient with its 95% confidence interval; β: standardized coefficient; *r*_*a(b.c)*_: semi-partial correlation;

* *p* < .05,

** *p* < .01

### 3.2 *fMRI activation*

**Fig. 1:**
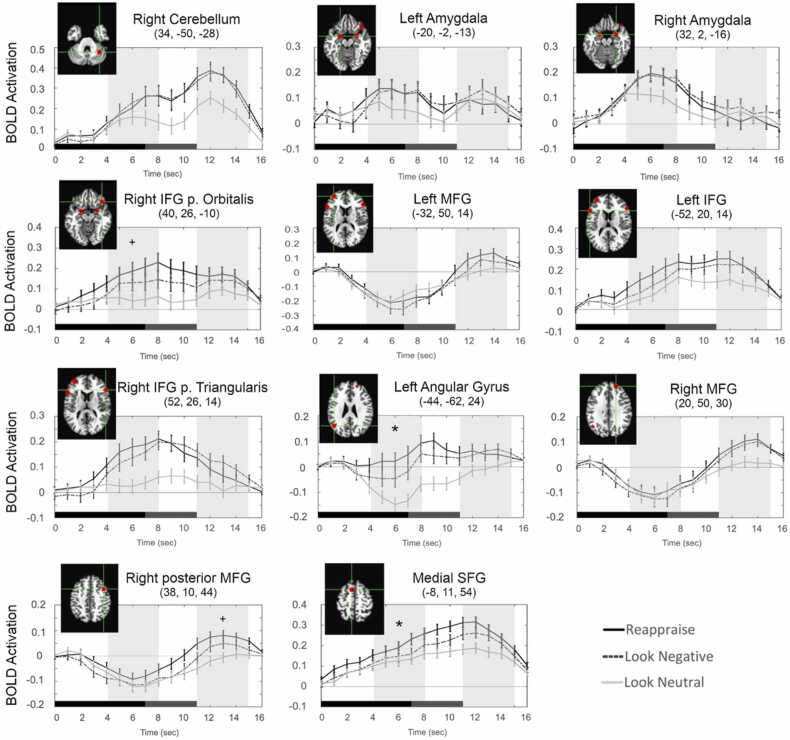
: Reappraisal-related brain activity in a priori regions of interest. HRF time courses extracted for each of the 11 ROIs for the three trial conditions: reappraise (black line), look negative (dashed line), and look neutral (gray line). The peak coordinate of each ROI (in Talairach space) is provided along with an image of its location on an anatomical brain slice. On each trial, the emotional image was presented for 7 s (black bar), followed by the rating screen for 4 s (dark gray bar). Light gray bands indicate the early (4–8 s) and late (11–15 s) windows from which the peak activation for reappraise minus look negative trials was calculated. *indicates a significant (^+^trend-level) difference from zero (reappraise – look negative) in that ROI during that time window (see Table 2). IFG: inferior frontal gyrus; MFG: middle frontal gyrus; SFG: superior frontal gyrus.

In the analysis of the HRF time courses from the 11 emotion regulation ROIs (Fig. 1), one sample *t*-tests on the average peak activation difference (reappraise – look negative trials) indicated two regions with significantly (Holm’s adjusted *p* < .05) increased activation in the early window (4–8 s post-stimulus onset): left angular gyrus and medial SFG, while activation in right IFG pars orbitalis approached significance (*p* = .091). Activation in the right posterior MFG ROI also approached significance (*p* = .055) in the late window (11–15 s post-stimulus onset; Table 2).

**Table 2:** One sample *t*-tests on ROI peak activation in early and late windows.

Time Window	ROI	Mean	SD	*t*	*Holm’s p*	Cohen’s *d*
Early	1) Right cerebellum	-0.0038	0.178	-0.185	1	-0.021
	2) Left amygdala	0.0096	0.164	0.507	1	0.058
	3) Right amygdala	-0.0018	0.151	-0.101	1	-0.012
	**4) Right IFG pars orbitalis**	**0.0621**	**0.205**	**2.637**	**0.091**^**+**^	**0.303**
	5) Left MFG	0.0394	0.170	2.026	0.307	0.232
	6) Left IFG pars triangularis	0.0511	0.192	2.325	0.182	0.267
	7) Right IFG pars triangularis	0.0324	0.150	1.884	0.317	0.216
	**8) Left angular gyrus**	**0.0580**	**0.173**	**2.914**	0.047 *	**0.334**
	9) Right MFG	0.0192	0.162	1.036	1	0.119
	10) Right posterior MFG	0.0298	0.127	2.050	0.307	0.235
	**11) Medial SFG**	**0.0420**	**0.119**	**3.082**	0.031 *	**0.354**
Late	1) Right cerebellum	0.0148	0.145	0.890	1	0.102
	2) Left amygdala	-0.0307	0.151	-1.776	0.478	-0.204
	3) Right amygdala	-0.0330	0.150	-1.919	0.412	-0.220
	4) Right IFG pars orbitalis	0.0288	0.226	1.111	1	0.127
	5) Left MFG	0.0488	0.208	2.042	0.357	0.234
	6) Left IFG pars triangularis	0.0123	0.192	0.559	1	0.064
	7) Right IFG pars triangularis	-0.0369	0.134	-2.407	0.176	-0.276
	8) Left angular gyrus	-0.0029	0.141	-0.177	1	-0.020
	9) Right MFG	0.0116	0.153	0.661	1	0.076
	**10) Right posterior MFG**	**0.0383**	**0.115**	**2.893**	**0.055**^**+**^	**0.332**
	11) Medial SFG	0.0347	0.125	2.426	0.176	0.278

One sample *t*-tests were two-sided versus zero and Holm’s adjusted *p*-values are given to correct for multiple comparisons. *p < .05, ^+^p < .10. IFG: inferior frontal gyrus; MFG: middle frontal gyrus; SFG: superior frontal gyrus.

**Fig. 2:**
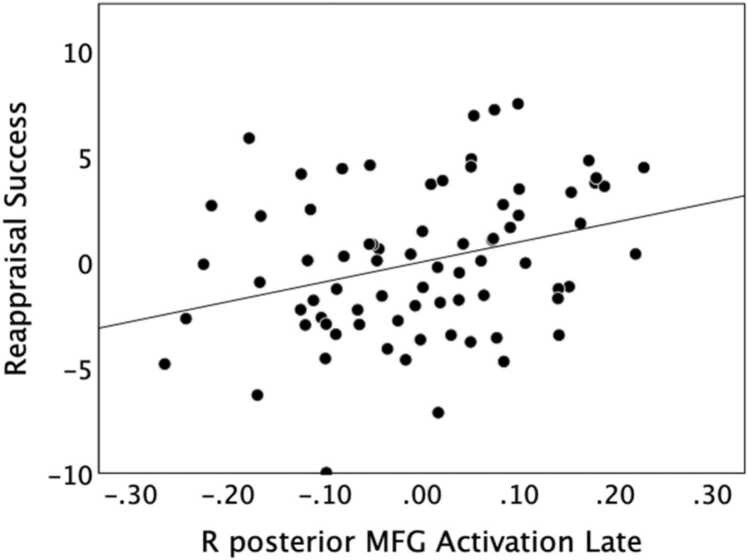
: Late activity in right posterior MFG supports reappraisal success. Correlation (controlling for age) between reappraisal success and increased peak activation in right posterior MFG during the late window for reappraise relative to look negative trials. Participants with greater average activation showed better reappraisal success.

**Fig. 3:**
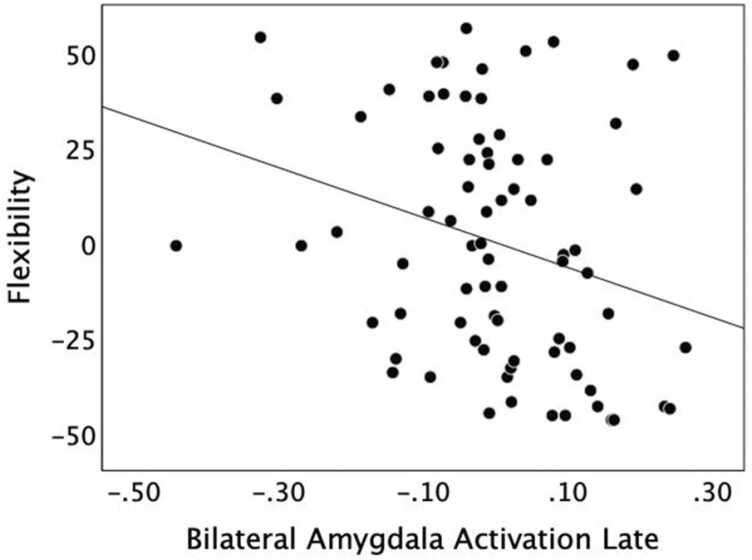
: Late decrease in amygdala activity supports flexibility. Correlation (controlling for age) between affective flexibility (ranked) and bilateral amygdala activation in the late window for reappraise relative to look negative trials. Participants with more reduced average activation for reappraise trials in left and right amygdala during the late window had higher affective flexibility scores.

Next, the behavioral measures of reappraisal success and affective flexibility were correlated with the peak activation in four averaged regions: the ROIs that showed significant or trend-level activation during 1) the early window (right IFG pars orbitalis, left angular gyrus, and medial SFG) or 2) the late window (right posterior MFG), as well as activation from the bilateral amygdala in the 3) early and 4) late windows. None of the averaged ROI activations correlated with age (all *p* > .05), but given the correlation between age and reappraisal success, these correlations with brain activation were controlled for effects of age. There was a significant positive correlation between reappraisal success and peak activation in the ROI that increased in the late window (*ρ* = 0.319, Holm’s adjusted *p* = .035; Fig. 2**;** Table 3), such that greater reappraisal-related activation in the right posterior MFG was associated with a greater reduction in reported negative affect (i.e., reappraisal success). There was also a significant negative correlation between affective flexibility and peak activation in the bilateral amygdala in the late window (*ρ* = −0.327, Holm’s adjusted *p* = .032; Fig. 3**;** Table 3), indicating that participants who were more flexible showed a greater reduction in amygdala activation late in reappraise trials. No other ROI activation or time windows were significantly correlated with the behavioral measures.

**Table 3:** Correlations of behavioral measures with average ROI peak activation in the early or late window, controlling for effects of age.

	1	2	3	4	5
1) Flexibility					
2) Reappraisal Success	0.187 [− 0.047,0.402]				
3) Increased Early ROIs	0.141 [− 0.094,0.361]	0.029 [− 0.204,0.259]			
4) Increased Late ROI	0.275^+^ [.046,0.476]	0.319 * [0.094,0.513]	0.205 [− 0.028,0.417]		
5) Bilateral Amygdala Early	-0.039 [− 0.268,0.195]	0.005 [− 0.227,0.236]	0.547 * [0.361,0.691]	0.130 [− 0.105,0.352]	
6) Bilateral Amygdala Late	-0.327 * [− 0.520, − 0.103]	-0.066 [− 0.293,0.169]	0.092 [− 0.142,0.318]	-0.025 [− 0.256,0.208]	0.265 [0.036,0.468]

Values for each pairing are the Spearman correlation (*ρ)* with its 95% confidence interval; * *p* < .05, ^+^*p* < .10. Increased early ROIs included the average of right IFG pars orbitalis, left angular gyrus, and medial SFG; the increased late ROI was right posterior MFG (see Table 2).

## 4 Discussion

In this study, children and adolescents ages 6–17 years old performed two tasks to assess their affective flexibility and cognitive reappraisal abilities. Affective flexibility and age predicted cognitive reappraisal success, along with a significant interaction which indicated that especially for children, more flexibility was associated with a greater regulation-related reduction in negative affect. With regard to fMRI activation of emotion regulation brain regions in early and late windows of the HRF time course, several ROIs had stronger activation during reappraise trials compared to look negative trials, consistent with previous emotion regulation studies of adults (Petro et al., 2018, Pierce et al., 2022). Notably, we also found evidence for dissociable reappraisal-related brain mechanisms that support reappraisal success and affective flexibility. Specifically, late increases in prefrontal cortex activity (right posterior MFG) supported reappraisal success, but late decreases in amygdala activity supported affective flexibility. Together, these results suggest that affective flexibility, as measured in our novel task, may support more mature behavioral and brain responses during cognitive reappraisal in children and adolescents, helping to build the affective skills that underlie this fundamental emotion regulation ability.

### 4.1 Affective flexibility predicts reappraisal success

As reported in an overlapping sample (Pierce et al., 2022), affective flexibility and age significantly predicted behavioral reappraisal success. Affective flexibility was operationalized as response variability in valence judgments of ambiguous facial expressions, whereas cognitive reappraisal success was derived from an emotion regulation task where participants were instructed to reinterpret emotional scenes to downregulate their negative feelings. Older participants were better at reappraisal than younger participants, consistent with previous reports and a broad developmental improvement in cognitive control (Crone and Steinbeis, 2017, Luna et al., 2015, McRae et al., 2012, Theurel and Gentaz, 2018). A significant interaction further revealed that specifically for children (ages 6–11), more affective flexibility in judgments of ambiguous stimuli translated to better (more mature) reappraisal performance. This pattern was not evident in the adolescent (ages 12–17) participants. Thus, it appears that affective flexibility may be a developmental stepping-stone to successful cognitive reappraisal that taps into a critical ability to create varying emotional appraisals. Flexibility in children may compensate for a lack of regulatory cognitive control of emotion, whereas adolescents may no longer rely upon affective flexibility only, potentially drawing on more developed cognitive abilities to overcome individual differences in this skill.

### 4.2 Reappraisal activation early and late

In the emotion regulation fMRI task, peak activation was significantly increased in two ROIs in the early window (left angular gyrus and medial SFG), with a trend-level increase in the right IFG pars orbitalis in the early window and the right posterior MFG in the late window. Generally, the pattern of activations across all ROIs was consistent with the original study of adults from which the ROIs were drawn (Petro et al., 2018), indicating that multiple regions of the PFC (and parietal cortex) support affective processing and cognitive reappraisal of negative emotional images in children and adolescents. Furthermore, the current HRF results paralleled those from a study of adults performing the same task (Pierce et al., 2022) in which both early and late peak activations were sensitive to reappraisal. Yet, as in Pierce et al. (2022), the regions that were activated and the strength of activation differed between the early and late windows of the time course, suggesting that MFG may support different affective functions that were engaged later in the trial than regions such as IFG and medial SFG that were activated early (see Section 4.3 below).

In addition to increased PFC activation during reappraisal, some previous studies of emotion regulation in adults (Buhle et al., 2014, McRae et al., 2012, Petro et al., 2018, Pierce et al., 2022) reported a significant decrease in amygdala activation. In the current results, however, no regions had significantly decreased activation during regulation, although bilateral amygdala and right IFG pars triangularis did tend towards reduced reappraisal activation during the late window. Across a wide range of tasks, the amygdala reacts to salient emotional stimuli (Cunningham and Brosch, 2012, Sander et al., 2003, Whalen, 1998), particularly those with negative valence (Costafreda et al., 2008). The reduced amygdala activation during emotion regulation, therefore, is often considered a physiological indicator of successful downregulation by PFC (Buhle et al., 2014, Cohen et al., 2016, Dörfel et al., 2014, Ochsner and Gross, 2008). Nonetheless, given that children and adolescents generally are more emotionally reactive and have worse cognitive control than adults (Gee et al., 2013, McRae et al., 2012, Silvers et al., 2015, Stephanou et al., 2016), the lack of significant amygdala deactivation in the current study is not unexpected. It is also possible that the timing of the amygdala response differs during development (cf. Dennis and Hajcak, 2009), and that the early and late windows applied in the current analysis do not capture the optimal peak (de)activation for all participants or ages.

### 4.3 Dissociable correlates of late reappraisal activation

In comparing brain responses with behavioral measures, we found that reappraisal success was positively associated with peak activation in an ROI that increased during the late window, the right posterior MFG. This region of PFC may exert control over the emotional response, direct attention to appropriate features of the stimulus, and maintain new appraisals in working memory until a decision on the emotion rating is made (Buhle et al., 2014, Ochsner et al., 2012). In our earlier study of emotion regulation in adults (Pierce et al., 2022), late activation in another PFC region, left MFG, was similarly associated with reappraisal success. This implies that children and adolescents are engaging similar brain regions as adults for the regulation task and individual differences in PFC recruitment similarly correspond to the effectiveness of cognitive reappraisal.

In addition to this behavioral correlate of PFC activation, late bilateral amygdala activation was negatively related to affective flexibility. Specifically, participants who were more flexible in interpreting ambiguous affective stimuli dampened amygdala activation to a greater extent during reappraise trials. Even though children and adolescents were not able to downregulate their amygdala response overall, greater affective flexibility did allow some individuals to achieve this physiological downregulation. Reduced amygdala activation is generally associated with a weaker emotional response or diminished salience of a stimulus (Buhle et al., 2014), which would indicate that the more flexible participants were implementing the emotion regulation task goals more effectively than less flexible participants.

It is worth noting that significant correlations were observed only for activation in the late window, which followed the presentation of the rating screen. This suggests that later activation may reflect the final reappraisal and affective evaluation of the stimulus more than early activation from the initial emotional response to the image presentation (see also Pierce et al., 2022). Affective flexibility may therefore relate more to slower processes during cognitive reappraisal or later processes related to emotion self-evaluation to generate a final rating, rather than to early, fast processes that occur soon after stimulus presentation. Because our flexibility task involves assessment of the valence of an emotional stimulus, this process may correspond most directly with the late, rating phase of the emotion regulation task when the participant must similarly assess the negativity experienced in response to the reappraised scene.

### 4.4 Affective flexibility as a building block for cognitive reappraisal

Considering that affective flexibility predicted both behavioral reappraisal success and a greater reappraisal-related reduction in amygdala activation, there is evidence that this ability to flexibly interpret ambiguous stimuli as positive or negative supports emotion regulation performance in development. Cognitive reappraisal necessitates the reinterpretation of emotional stimuli via recruitment of the PFC, guiding affective appraisals away from an initial negative reaction towards a more neutral or positive viewpoint (in accordance with the current goal of downregulation of negativity; Ochsner et al., 2012). It follows, therefore, that this reinterpretation ability and the corresponding downregulation of amygdala are related to flexibility in interpretations of ambiguous emotional stimuli (cf. Malooly et al., 2013). Interestingly, the behavioral effect was most evident in the children in our sample (rather than the adolescents) in whom additional reappraisal skills that depend on cognitive control are likely to be less fully developed. Thus, this type of flexibility may provide affective scaffolding to support the formation and self-evaluation of less negative reappraisals during emotion regulation in younger individuals who otherwise may not possess the necessary attentional or inhibitory control mechanisms to overcome their initial (negative) emotional response.

### 4.5 Limitations and conclusion

The current results should be considered with some limitations. First, in the emotion regulation task the image and rating screen were temporally locked and activation during the late window could arise from slow activation in response to the image itself or from a response specific to the rating screen. Future work using jittered presentation or trials without a rating screen is necessary to disambiguate the contribution of the various trial stages and task processes that may contribute to the early and late window activations. Another limitation is that the ROIs used in this study were derived from a study of young adults. It is therefore possible that some regions which are relevant to affective flexibility but show developmental differences may not have been considered here, although there is evidence that children and adolescents do engage similar regions as adults during emotion regulation (McRae et al., 2012). A whole brain analysis that focuses on simple activation rather than HRF shape and timing could prove useful in exploring other regions potentially related to affective flexibility.

In conclusion, this study provides a novel measure of affective flexibility that is developmentally appropriate and overcomes previous measures’ reliance on cognitive control. Notably, this work lays the critically needed foundation that establishes this measure as a mechanism supporting cognitive reappraisal in development. Broadly, the ability to flexibly respond to changing environmental conditions leads to better psychological outcomes (Kashdan and Rottenberg, 2010, Uddin, 2021) and resilience in the face of adversity (Parsons et al., 2016). Therefore, children who develop better affective flexibility skills may show better emotion regulation over time and less vulnerability to mental health disorders. Longitudinal studies of the relationship between affective flexibility, cognitive reappraisal ability, and various psychological outcomes are needed to explore this possibility.

### Ethics approval

All study procedures were approved by the UNL Institutional Review Board; all children provided verbal assent with parent/guardian written consent.

## Funding

This work was supported by the 10.13039/100000002National Institutes of Health (NIMH111640; PI: Neta), and by a 10.13039/100000001National Science Foundation CAREER award (PI: Neta).

## CRediT authorship contribution statement

**Jordan E. Pierce:** Formal analysis, Writing – original draft, Writing – review & editing. **Eisha Haque:** Data curation, Investigation, Writing – review & editing. **Maital Neta**: Conceptualization, Funding acquisition, Methodology, Resources, Supervision, Writing – review & editing.

## Declaration of Competing Interest

The authors declare that they have no known competing financial interests or personal relationships that could have appeared to influence the work reported in this paper.

## Data Availability

This study was not preregistered. The task data are available online at https://osf.io/e7ugk/.
